# A Predictor Combining Clinical and Genetic Factors for AML1-ETO Leukemia Patients

**DOI:** 10.3389/fonc.2021.783114

**Published:** 2022-01-14

**Authors:** Min Yang, Bide Zhao, Jinghan Wang, Yi Zhang, Chao Hu, Lixia Liu, Jiayue Qin, Feng Lou, Shanbo Cao, Chengcheng Wang, Wenjuan Yu, Hongyan Tong, Haitao Meng, Jian Huang, Honghu Zhu, Jie Jin

**Affiliations:** ^1^ Department of Hematology, The First Affiliated Hospital, College of Medicine, Zhejiang University, Hangzhou, China; ^2^ Institute of Hematology, Zhejiang University, Hangzhou, China; ^3^ Zhejiang Province Key Laboratory of Hematology Oncology Diagnosis and Treatment, Hangzhou, China; ^4^ Department of Hematology, The Second Affiliated Hospital of Xi’an Jiaotong University, Xi’an, China; ^5^ Acornmed Biotechnology Co., Ltd., Beijing, China

**Keywords:** acute myeloid leukemia, AML-ETO, next-generation sequencing, prognosis, LASSO Cox regression

## Abstract

Core Binding Factor (CBF)-AML is one of the most common somatic mutations in acute myeloid leukemia (AML). t(8;21)/AML1-ETO-positive acute myeloid leukemia accounts for 5-10% of all AMLs. In this study, we consecutively included 254 AML1-ETO patients diagnosed and treated at our institute from December 2009 to March 2020, and evaluated molecular mutations by 185-gene NGS platform to explore genetic co-occurrences with clinical outcomes. Our results showed that high KIT VAF(≥15%) correlated with shortened overall survival compared to other cases with no KIT mutation (3-year OS rate 26.6% vs 59.0% vs 69.6%, HR 1.50, 95%CI 0.78-2.89, P=0.0005). However, no difference was found in patients’ OS whether they have KIT mutation in two or three sites. Additionally, we constructed a risk model by combining clinical and molecular factors; this model was validated in other independent cohorts. In summary, our study showed that c-kit other than any other mutations would influence the OS in AML1-ETO patients. A proposed predictor combining both clinical and genetic factors is applicable to prognostic prediction in AML1-ETO patients.

## Introduction

The t(8;21)(q22;q22) is the most commonly observed chromosomal translocation in acute myeloid leukemia (AML) patients; it generates the AML1-ETO (AE) fusion protein (1–4). Adult AMLs with AML1-ETO account for approximately 5%–8% (5–7) and are associated morphologically with AML-M2/M4 subtypes. The median age of these patients is considerably lower, and the prognosis is better compared to normal-karyotype AMLs or other chromosome aberrations. This favorable consequence is associated with a higher complete remission (CR) rate and lower relapse incidence (8–10). However, some subtypes of AML1-ETO-postive AML patients were observed with a higher incidence of relapse and poorer outcomes, and the coexisting c-kit activation mutations may be one of the underlying reasons. Adult patients with CBF leukemia had 12.8%–46.1% of c-kit mutations (11–13). Whether or not other genetic mutations will cooperate with AML1-ETO to drive disease progression is still unknown. We do not know, for example, if oncogene mutations such as ASXL2, KMT2A, and TET2 in CN-AMLs have any biological influence in AML1-ETO-postive AML or if the co-occurrences of driver genes with AML1-ETO would contribute to poor clinical outcomes. In fact, it has been reported that mutations in coding driver genes determined by next-generation sequencing (NGS) technology, such as c-kit, cause other molecular disorders that lead to myeloid leukemia. Additionally, the widely established genetic factors can predict clinical outcomes for newly diagnosed AML1-ETO-postive AMLs. Therefore, it is urgently needed to further study the characteristics of the AML1-ETO molecular, particularly in assessing their accompanied genes and their more potential prognostic markers.

Although previous studies have observed poor prognostic roles of the concomitant gene with KIT (9, 10), the impact of the specific mutation sites cooperated with their concomitant genes for the Chinese population, and the most important co-mutations with distinct prognosis in AML1-ETO patients are still controversial. Meanwhile, whether or not some critical co-mutations may translate into treatment decisions in clinical practice still requires investigation. According to European Leukemia Net (ELN), AML1-ETO patients with c-kit mutation tend to have an unfavorable prognosis with higher relapse rates. However, there are various mutation sites, and we do not know which one is the key (14). It is also reported that AML1-ETO patients with c-kit of EV8-11 may suffer from poor outcomes (15). In summary, the aforementioned studies implied that more co-mutations with prognostic significance remain to be discovered.

In recent years, NGS has been used in genomic and epigenetic research with the advantage of high throughput, high sensitivity, and high stability. However, there still lacks uniformed statement about how to precisely identify the risk stratification of patients with AML1-ETO, especially combining with the results of NGS. Herein, we conducted a study of 254 AML1-ETO-positive AML patients to explore the genetic profiling and identify the co-mutated genes with distinct prognosis. In summary, the aim of this study is to construct an easily applicable complex gene model for overall survival (OS) in patients with newly diagnosed AML1-ETO-positive AML.

## Methods

### Cases

**Figure 1:**
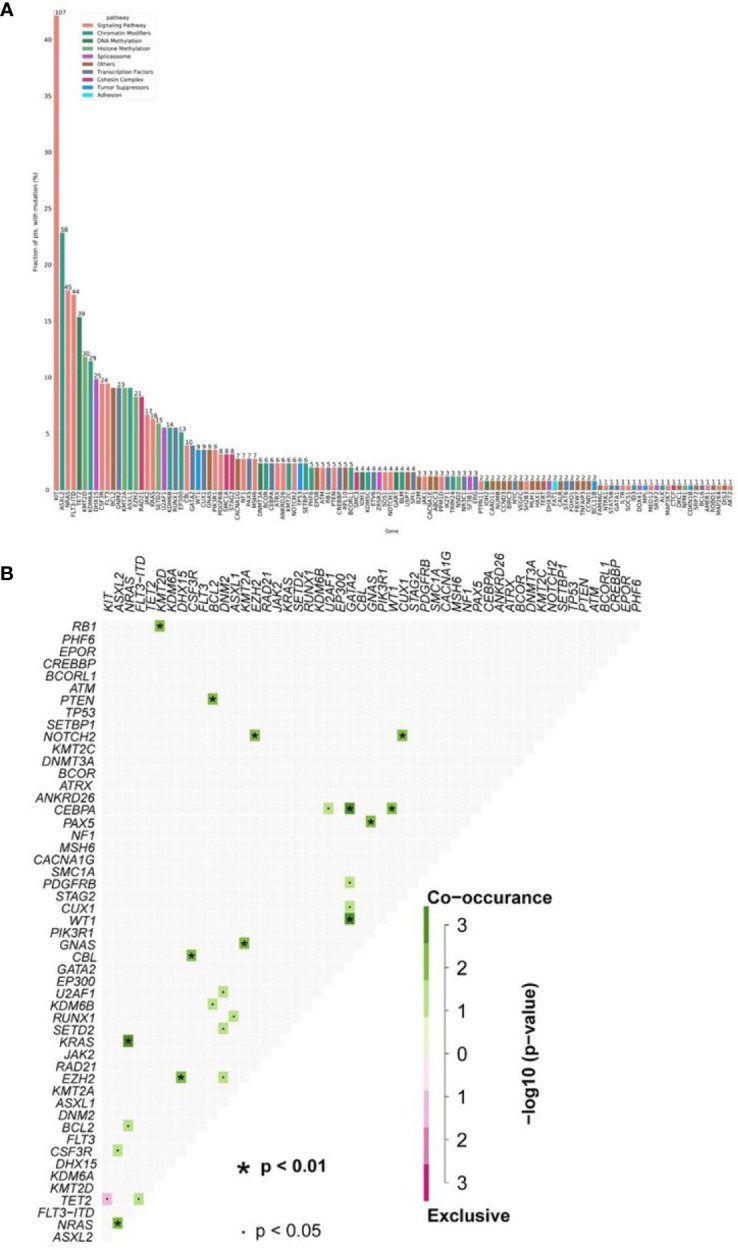
**(A) **Mutational landscape in 254 AML1-ETO patients. **(B)** The number of gene mutations per patient. (Each row represents stated gene; each column represents a patient; the right side of the graph annotates the frequency and number of the gene; the upper histogram showed the number of gene mutations per patient; different colors below the graph represent different mutation patterns).

This study included 254 patients with *de novo* AML with AML1-ETO-positive, who were diagnosed between December 2009 to March 2020 and treated in the First Affiliated Hospital of Zhejiang University (**Figure 1**). Patients who died before induction or receiving non-standard dose induction therapies were excluded. All patients were informed about the study and provided written consents following the Declaration of Helsinki. The study was approved by the ethics committee of the First Affiliated Hospital of Zhejiang University. Diagnosis of AML was determined in accordance with the 2016 revision of World Health Organization (WHO) classification of myeloid neoplasms and acute leukemia (16). Cytogenetic risks were classified based on the updated risk stratification and management (17). All 254 patients received induction therapy. Among them, 70.9% (n = 180) received IA 3 + 7 regimen [idarubicin 8–10 mg/m^2^ for 3 days plus cytarabine (Ara-C) 100 mg/m^2^ for 7 days], 12.2% (n = 31) received demethylation treatment [decitabine 10 mg/m^2^ for 3 days plus low-dose chemotherapy (Ara-C) 20 mg/m^2^ for 14 days], and 9.8% (n = 25) received HAA regimen (homoharringtonine 2 mg/m^2^, Ara-C 100 mg/m^2^, and aclarubicin 20 mg/day for 7 days). Patients who failed to achieve CR after first induction were treated by reinduction (same regimen if partial remission after the first cycle, crossed regimen if no remission). Patients who relapsed and had suitable donor underwent allogeneic hematopoietic stem cell transplantation (allo-HSCT).

### Next-Generation Sequencing Experiment Design and Methods

NGS was performed in all 254 patients using a panel of 185 genes, which covered all the mutation hotspots of acute leukemia (AL), myelodysplastic syndrome (MDS), and myeloproliferative neoplasms (MPNs) (**Supplementary Table S1**). The gene chip was provided by Acornmed Biotechnology Co., Ltd. Genomic DNAs were extracted from mononuclear cells isolated by Ficoll gradient centrifugation from bone marrow (BM) samples at primary diagnosis by DNeasy 96 Blood & Tissue Kit (Qiagen, Hilden, Germany). Gene library amplification was based on KAPA Library Amplification Kit (KAPA, Boston, MA, USA). Roche NimbleGen kit (Roche, Basel, Switzerland) was used to capture the target region. The hybridized captured samples were sequenced using NovaSeq 6000 PE150.

Preprocessing of raw sequence data and quality control statistics were performed by using an indigenous QC program. Reads were aligned to the hg19 version of the human genome using Burrows–Wheeler Alignment tool (BWA, version 0.7.12). PCR duplicates were marked using the MarkDuplicates tool in Picard. IndelRealigner and BaseRecalibrator on Genome Analysis Toolkit (GATK; version 3.8) were used to conduct realignment and recalibration of the BWA alignment results, respectively. Mutect2 was used for identifying paired-sample variant calling of SNV and INDEL. We obtained candidate variations through background database filtering of normal samples. Pindel (version 0.2.4) (13) was used for detecting CBF. We also compared the quantitative results with electropherogram to ensure the reliability of NGS. All the variants were annotated by ANNOVAR software.

Multiplexed libraries were sequenced using 150-bp paired-end runs on an Illumina Novaseq. To ensure the quality of data, the following criteria were performed to filter raw variant results: Average effective sequencing depth on target per sample ≥1,000x; Allele mutation frequency ≥1% for single-nucleotide variation and insertion or deletion, respectively; all reads were filtered by high Mapping quality (≥30) and Base quality (≥30); the mutant reads need to be supported by positive and negative strands. Furthermore, we excluded synonymous variants, as well as Single Nucleotide Polymorphisms (SNPs) when reported in the 1000 Genomes Project database (October 2014 release) at a population frequency >1% or our in-house SNP databases.

### Definition of Clinical End Points

CR was defined as less than 5% bone marrow blasts and showing normal maturation of all cell lineages, no blast in peripheral blood, absolute neutrophil count >1.0 × 10E9/L, platelet count (PLT) >100 × 10E9/L, and no extramedullary leukemia. Early death (ED) referred to all causes of death within 30 days from the first day of induction chemotherapy. Relapse was defined as more than 5% blasts in bone marrow, reappearance of blasts in peripheral blood, or extramedullary leukemia in patients with previously documented CR. OS was defined as the time from diagnosis to death or last follow-up. Relapse-free survival (RFS) was defined as the time from first CR to relapse, censoring at death in CR or last follow-up. The last follow-up was done on December 5, 2020.

### Statistical Analysis

Data analysis was performed with SPSS (Version 25) and R (version 3.6.3). Differences in continuous variables were analyzed by Mann–Whitney *U*-test, and categorical variables were compared by chi-square test or Fisher’s exact test. OS and RFS probabilities were estimated using the Kaplan–Meier survival analysis, and the differences in survival curves were compared by log-rank test. Cox hazard models were used to assess the clinical variables with survival. Factors for univariate analysis included clinical characteristics [sex, age (median 38 years)], laboratory characteristics [white blood cells (WBCs) (median 17.9 × 10^9^/L), hemoglobin (Hb) (median 79 g/l), PLT (median 40 × 10^9^/L), lactate dehydrogenase (median 757 U/L), bone marrow (BM) blasts (median 48%)], and AML1-ETO molecular characteristics [VAF (0.48%), AML1-ETO number (1 or more than 1), length (bps), location (JM/beta1-sheet/other domains)]. A statistical significance level of 0.1 in the univariate analysis was used to select variables in the risk score system. Least Absolute Shrinkage and Selector Operation (LASSO) Cox regression model was used in variable selection and predictive prognostic model construction. A two-tailed p < 0.05 was considered significant.

## Results

### Clinical Characteristics of the AML1-ETO-Positive Patients at Diagnosis

Patients’ characteristics and additional molecular and cytogenetic aberrations are shown in **Table 1** according to CBF subtype. The median follow-up was 4.5 years.

**Table 1:** Characteristics of 254 AML-ETO patients.

Variable	Number (%)/Median (range)
Sex	
Male	140 (57.9)
Female	102 (42.1)
Age, years, median (range)	38 (10–78)
>60 years	18 (7.4)
WBC, 10^9^/L	10.5 (1.3–192.7)
≥100 × 10^9^/L	8 (3.3)
Hb, g/L	55.5 (32–160)
PLT, 10^9^/L	26 (3–300)
LDH, U/L	517 (106–6,545)
>245	52 (21.5)
Bone marrow blasts, %	40.3 (20–91.5)
ECOG	
0	82 (33.9)
1	89 (36.8)
2	20 (8.3)
FAB	
M0	0 (0)
M1	0 (0)
M2	179 (74.0)
M4	25 (10.3)
M5	36 (14.9)
M6	0 (0)
MPAL	2 (0.8)
Karyotype	
Normal karyotype	20 (8.7)
t(8;21)(q22;q22)	210 (86.8)
HSCT	66 (27.3)

WBC, white blood cell; Hb, hemoglobin; PLT, platelet count; LDH, lactate dehydrogenase; ECOG, Eastern Cooperative Oncology Group; FAB, morphology according to French–American–British classification; MPAL, mixed phenotype acute leukemia; HSCT, hematopoietic stem cell transplantation.

Of the total 254 patients, the median age was 38 (10–78) years and 41.7% (106/254) were male. AML1-ETO was most common in M2 (68.9%, 175/254), second in M5 (15.0%, 38/254) (**Table 1**). Consistent with previous findings (7, 14), high WBC count (median 17.9 × 10E9/L, 55.1% over 100 × 10E9/L) and high percentage of BM blasts (median 48.0%, range 3.0%–91.5%) were observed. Among all the patients, 11.8% (30/254) had normal karyotypes, 5.1% (13/254) had -X chromosomes, and 11.0% (28/254) had -Y chromosomes. CRs were achieved in 85.0% (216/254) patients, and 27.6% (70/254) relapsed after CR.

**Figure 2:**
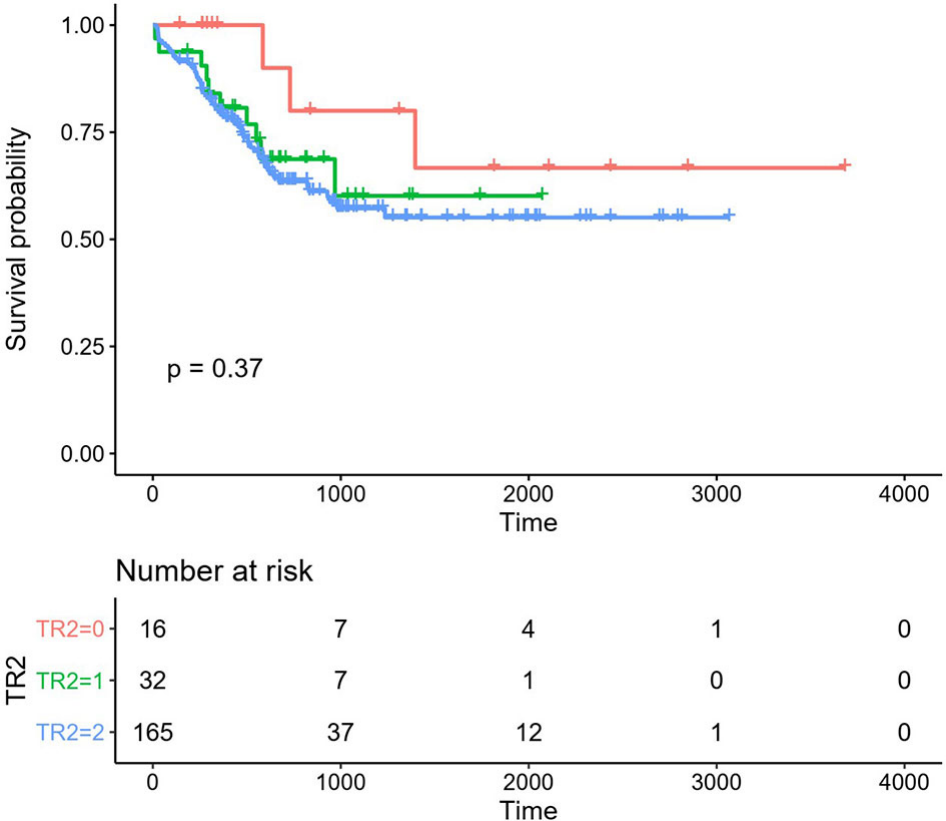
Overall survival (OS) in the different regimen patient groups. Red line: HAA regimen, 3-year OS 80.00% ± 12.65%; Green line: HAA/decitabine regimen, 3-year OS 60.12% ± 11.13%; Blue line: IA regimen, 3-year OS 57.21% ± 4.58%.

The total number of patients of the HAA group is lower than that of the IA group. The CR rates were different between IA group and HAA group (p = 0.01). Early deaths occurred in 5.1% (13/254) patients. The median follow-up was 53.7 (6–121) months for all patients. Median OS was 11.4 (95% CI 7.8–15.0) months, and median RFS rate was not reached; OS rate at 3 years was 28.4% ± 6.0%, and RFS rate at 3 years was 57.8% ± 8.0%. In addition, 40.8% (20/49) patients underwent HSCT in CR1, which showed significantly superior outcomes than those who did not (OS p = 0.002, RFS p = 0.015, respectively). Details of the patients were summarized in **Tables 1** and **2**. The OS of HAA plus decitabine regimen was superior than that of IA regimen, but there is no significant difference between HAA regimen and decitabine regimen. Due to the limited number of patients with c-kit gene (3 out of all 31 patients) receiving decitabine regimen (**Figure 2**), it is not feasible for us to take further evaluation.

**Table 2:** Patient enrollment flowchart.

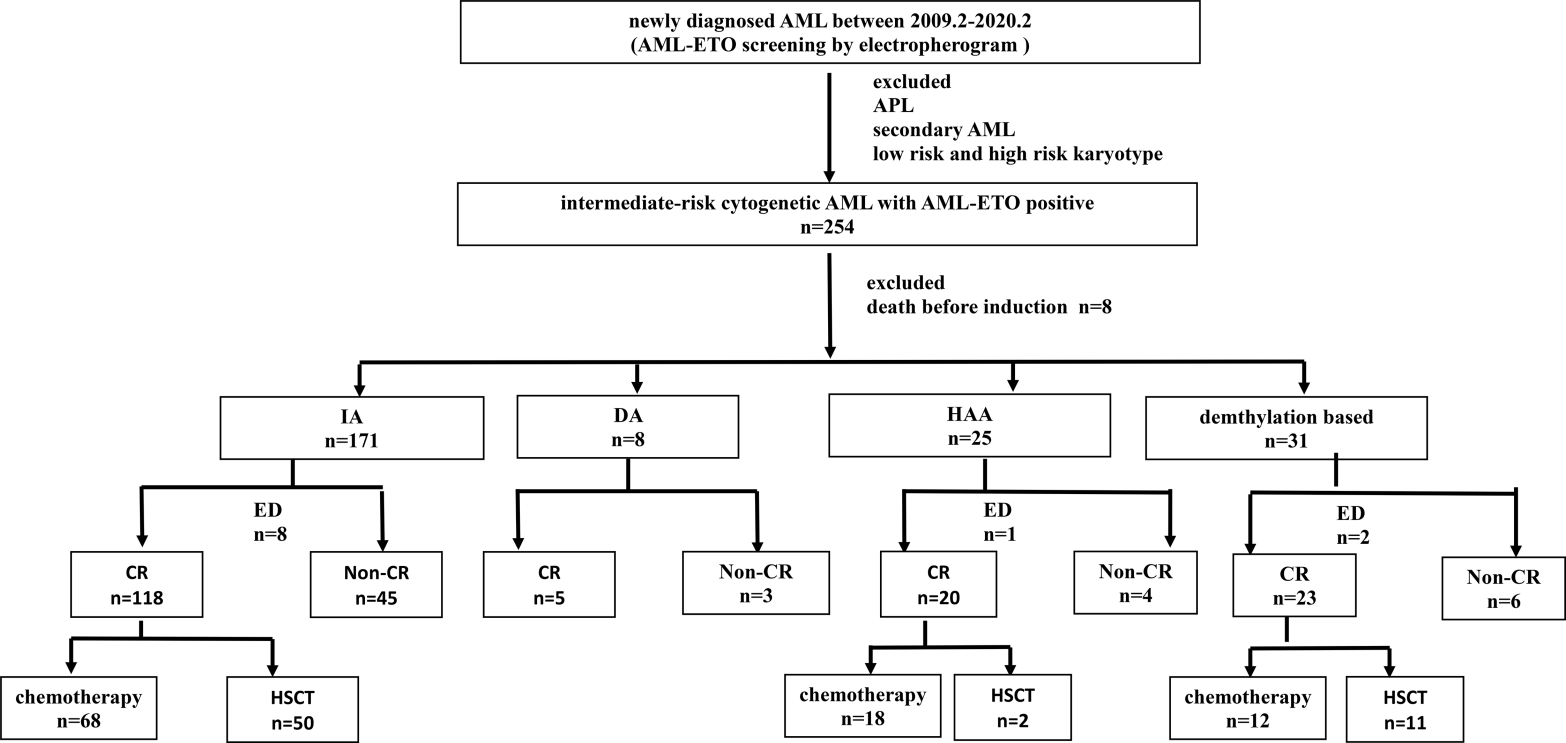	

### Mutational Landscape of AML1-ETO-Positive Patients

Thirty-seven different mutant genes were detected in the 254 patients, and the details of mutations were presented in **Figure 1**. The top five common concomitant mutations were KIT (42%), ASXL1 (23%), NRAS (17.7%), FLT3-ITD (17.3%), and TET2 (15.3%). The median number of co-mutant genes was 3 (0–10), but the number of mutations did not affect the OS and RFS except kit (both p > 0.05). For genes detected in more than 3 patients, we performed gene association analysis and found interesting coexistence and mutual exclusion relationships (**Figure 1**). For example, kit, *FLT3-ITD*, and *WT 1,IDH2* tend to appear together, while *NPM 1-WT1* and *NPM 1–RUNX1* appear to be mutually exclusive (p = 0.00008; p = 0.0014, respectively). Here, 132 patients (29.1%) have 2 or more mutations/fusion genes (18). In our study, the frequencies of concomitant genes were WT1 24.4% (62/254) FLT3-ITD 17.3% (44/254). There was a study that showed that mutations of c-kitD835 were associated with poor prognosis and elevated WBCs, especially in patients with inv(16) (19). C-kitD816 was the most common mutation in our study; the frequency was 54% (58/107) in all the c-kit mutation patients. These mutations mostly occur in exon 8 or 17 and are observed in 25% AML1-ETO cases (11). We found 124 KIT mutations in 84 patients, of which 91 mutations were located in exon 17 (73%), mainly SNVs at D816 or N822. In exon 8, we found 24 mutations (19%), mainly in-frame insertions/deletions at positions 416 to 422.

### The Numbers of c-kit Concomitant Mutations

**Figure 3:**
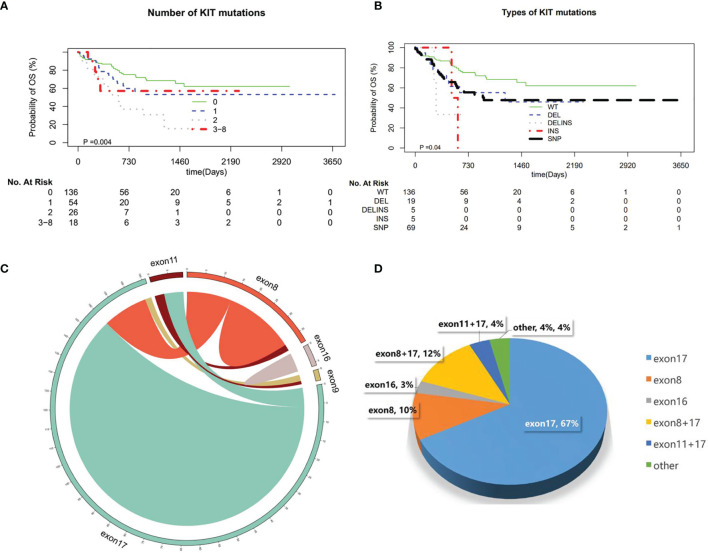
**(A)** Number of KIT mutations. **(B)** Types of KIT mutations. **(C)** The distribution of KIT exons. **(D)** The scale of KIT exons.

C-kit mutations are significant prognostic predictors in CBF-AML patients with t(8;21) and inv(16), which are associated with poor prognosis; however, current findings are inconsistent in this regard (20, 21). Nevertheless, different OS rates have been reported for CBF-AML patients with c-kit mutations compared to others (6) (**Figure 3**). Current research still has inconsistent conclusions regarding whether c-kit is related to poor prognosis, while our results had clearly shown a relationship.

In our study, the frequency of c-kit concomitant genes were 42.0% (107/254), which is the same as in other studies (22). In the patients with c-kit mutations, the one-site mutation was most common. Two sites were rare. There were also 3–8 sites of c-kit mutations. We analyzed the patients with c−KIT mutation into exon 8, 17 and exon 10 groups; the numbers of the mutation had no effect on OS. There is no significant difference in OS whether there are two or three mutation sites. And for the mutation form of c-kit, insertion or deletion has no obvious impacts on OS. Although the mutation mode of INS in our study has a statistical difference in survival, considering the small sample size, it needs a large cohort of patients to further confirm the result.

### Molecular Characteristics of the AML1-ETO

**Figure 4:**
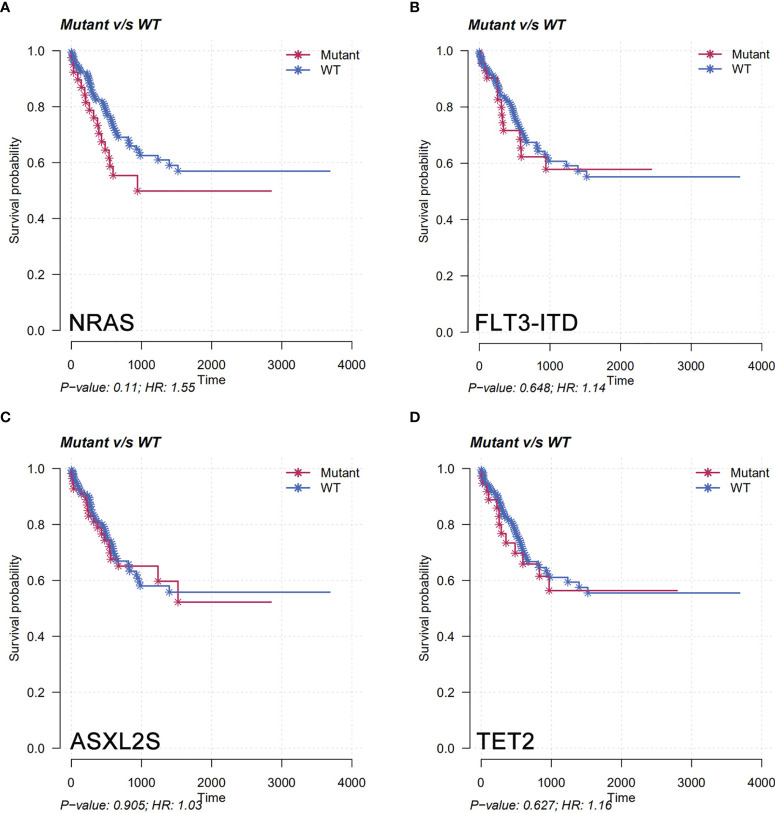
Overall survival (OS) and recurrence-free survival (RFS) in the entire patient group according to different molecular characteristics of AML1-ETO. **(A)** Mutation with NRAS. **(B)** Mutation with FLT3-ITD. **(C)** Mutation with ASXL2S. **(D)** Mutation with TET2.

The molecular structure characteristics of the AML1-ETO were summarized in **Figure 1**. The OS and RFS according to different molecular characteristics of AML1-ETO were shown in our results (**Figure 4**). Except for c-kit mutation, there is no significant difference in the OS of patients regardless of the genetic mutations.

The frequency of FLT3-ITD concomitant gene was 17.3% (44/254). The FLT3-ITD is a gene suggested with poor prognosis in AML, especially the FLT3-ITD mutation of higher frequency is an important factor affecting survival. In our study, the proportion of patients with higher frequency of FLT3-ITD mutations was suggested in AML1-ETO patients, while there is no impact on the OS in AML1-ETO patients, regardless of whether they are accompanied by FLT3-ITD mutation. The same results were also shown in AML1-ETO patients with ASXL2, NRAS mutation. That is different from the study of Duployez et al. (15). According to their results, the association of mutations remained associated with the highest hazard of relapse in patients with t(8;21) AML (15). C-kit, ASXL2, NRAS, and FLT3-ITD mutations were most common in our cohort. The OS showed no difference in AML1-ETO patients with these mutations.

### A Risk Score Combined Clinical and Molecular Profiles

Patients (n = 75) from the First Affiliated Hospital of Soochow University and Ningbo First Hospital were used as a validation cohort. The median age in the analyzed patients was 42 (6–78) years. The most common recurrent mutations occurred in KIT (n = 84, 50%), ASXL2 (n = 46, 28%), NRAS (n = 37, 22%), FLT3-ITD (n = 35, 21%), and TET2 (n = 30, 18%). We observed that high KIT mutant allele burden predicts poor outcome in t(8:21) AML. High KIT VAF (≥15%) correlated with shortened OS compared to the other KIT mutated cases including low VAF and wild-type KIT [3-year OS 26.6% vs. 59.0% vs. 69.6%, hazard ratio (HR) 1.50, 95% CI 0.78–2.89, p = 0.0005]. In addition, we also identified that some other mutated genes influence the prognosis of patients with t(8;21), such as FLT3-ITD high mutation burden (VAF ≥44% vs. other cases, 3-year OS 30.0% vs. 56.2%, HR 2.94, 95% CI 0.43–20.18, p = 0.056), TET2 high mutation burden (VAF ≥43% vs. other cases, 3-year OS 33.3% vs. 56.5%, HR 2.87, 95% CI 0.66–12.46, p = 0.018), and DHX15 high mutation burden (VAF ≥22% vs. other cases, 3-year OS 15.0% vs. 58.3%, HR 2.65, 95% CI 0.81–8.73, p = 0.011).

**Figure 5:**
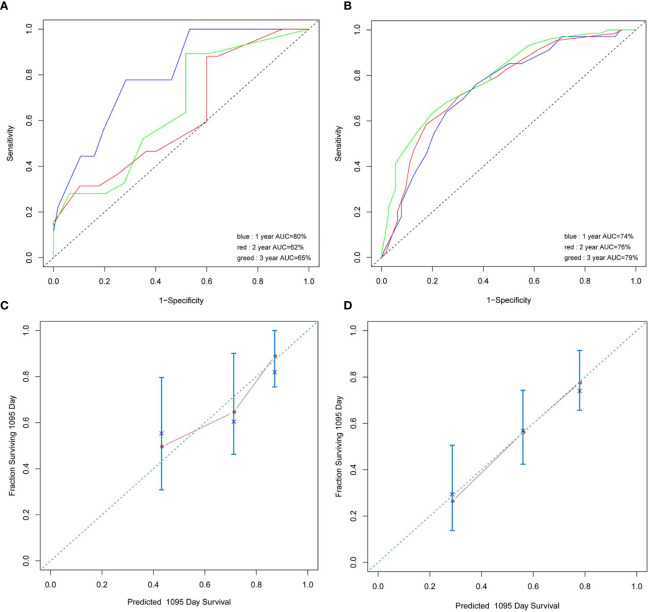
**(A, B)** Concordance index showed the risk score efficacy. **(C, D)** The calibration plots showed that the nomograms did well compared with an ideal model.

In univariate analyses for OS, age >42 years (3-year OS 46.3% vs. 64.4%, HR 1.91, 95% CI 1.14–3.14, p = 0.012), WBC >27.1 × 10^9^/L (3-year OS 34.3% vs. 60.0%, HR 2.59, 95% CI 1.13–5.9, p = 0.001), BM blast >20% (3-year OS 52.2% vs. 92.8%, HR 6.36, 95% CI 2.7–14.97, p = 0.035), LDH >504 U/L (3-year OS 44.1% vs. 67.1%, HR 2.62, 95% CI 1.50–4.59, p = 0.0007), PLT ≤28 × 10^9^/L (3-year OS 47.1% vs. 66.9%, HR 1.89, 95% CI 1.13–3.17, p = 0.019), and Hb ≤87 g/L (3-year OS 49.4% vs. 73.8%, HR 2.20, 95% CI 1.27–3.84, p = 0.019) were significantly associated with poor OS. Six variables were incorporated in our scoring model by LASSO, including age, WBC, PLT, KIT mutation, FLT3-ITD mutation, and TET2 mutation. A risk scoring model was developed by incorporating the weighted coefficients of these variables. The risk score grouped AML1-ETO AML patients into two subgroups: low risk (LR; n = 68) and high risk (HR; n = 86) groups. The 3-year OS rates for LR and HR groups were 72.7% and 43.0% (p < 0.0001; **Figure 5**). Similar results were also observed in the validation cohort (3-year OS 79.1% vs. 49.5%, p = 0.01; **Figure 5**). Concordance index (train: 0.708, 95% CI 0.680–0.736; validation: 0.722, 95% CI 0.666–0.778) demonstrated discrimination power well, and calibration plots showed that the nomograms did well compared with an ideal model. Six variables were incorporated in our scoring model by LASSO, including age, WBC, PLT, KIT mutation, FLT3-ITD mutation, and TET2 mutation. A risk scoring model was developed by incorporating the weighted coefficients of these variables. The risk score grouped AML1-ETO AML patients into two subgroups: LR (n = 68) and HR (n = 86) groups. The 3-year OS rates for LR and HR groups were 72.7% and 43.0% (p < 0.0001; **Figure 5**). Similar results were observed in the validation cohort (3-year OS 79.1% vs. 49.5%, p = 0.01; **Figure 5**).

## Discussion

In this study, we analyzed the gene distribution of AML1-ETO patients by NGS platform. It was concluded that Chinese AML1-ETO patients had many coexisting gene mutations, and the interaction was very complicated. Meanwhile, we explored the structural characteristics of AML1-ETO of each patient by NGS platform and found that the number of C-kit had significant effects on prognosis. Since multiple factors could affect the prognosis, we propose, for the first time, gene mutations of a large number of AML1-ETO-positive patients to predict patient outcomes. The databases from this study may be helpful for further investigation of the risk stratification and prognostic prediction regarding patients with AML1-ETO AML.

It is believed that the development of AML is a multistep process, which requires at least two genetic abnormalities for the development of the disease (17). We analyzed the gene expression of the 254 *de novo* AML1-ETO patients by target sequencing and discovered that most of the co-mutations clustered in methylation-related genes, chromatin-modifying genes, and transcription factor genes. C-kit, *ASXL2*, *NRAS*, and *FLT3-ITD* mutations were most common, while mutations such as *Bcl-2* and *KMT2A* were rare. The distribution according to gene function was similar to reports by Yu et al. and Duployez et al. using NGS, but the *contaminant* mutations without c-kit did not influence OS in our study (15, 22). Duployez et al. (15) showed that among the 215 patients analyzed, 182 (85%) had at least 1 mutation. In our study, the main mutation genes were c-kit (42%), ASXL2 (23%), NRAS (17.7%), and FLT3-ITD (17.3%). This is consistent with findings in the existing literature. While Duployez et al. (15) showed that diverse cooperating mutations may influence CBF-AML pathophysiology as well as clinical behavior and point to potential unique pathogenesis of t(8;21) vs. inv(16) AML, our study looks at AML-ETO patients in general, with an emphasis on the gene mutational landscape and the risk score combined clinical and molecular profiles, which can be a prognosis suggestion for AML-ETO patients.

Our results are similar with those in the Hong Kong center. More importantly, our study included the largest number of patients. Some studies showed that the most common mutation types combined with AML1-ETO patients are C-KIT, NRAS, and FLT3. Among all combined mutations, C-KIT has a poor prognosis and NRAS is meaningless. Our research is consistent with this result. Our study also found that FLT3-ITD in all patients did not affect the survival of AML1-ETO patients if the mutation load is not considered. When we proposed a threshold value, we found that 44% can be used as the value for the mutation load. The patients above this value correlated with poorer OS. This result can be used as the FLT3-ITD with AML1-ETO mutation threshold for Chinese people.

CBF-AMLs are commonly associated with favorable prognosis; however, this prognosis can be changed. With some kinds of gene mutations, correspondingly, only 50% of CBF-AML patients are able to preserve long-term remission without any relapse. Our results showed that the 3-year OS of HAA group was 80.00% ± 12.65%, while that of the IA group was 57.21% ± 4.58%. We proposed that the HAA regimen had more survival benefits than IA regimen, which was consistent with the result of Cao et al. (23). Seyhan et al. showed that RAS genes and identified their association with doxorubicin and etoposide sensitivity. They also found that GF2R, CTSA, and ATP6AP2 were gene biomarkers, which can subgroup AML patients into distinct good and bad prognostic groups (24). We try to find more drug-sensitive biomarker genes for AML-ETO patients and improve AML patient efficiency.

Previous studies have shown that AML1-ETO combined with FLT3-ITD would affect the survival of patients; however, our results suggested that the combination of FLT3-ITD did not affect the OS in this kind of patients. Our study included 254 AML1-ETO-positive AML patients. Although our study is a retrospective one, the follow-up time is long. It is also a study with a larger number of cases included.

The results of several studies showed the gene file of AML1-ETO and some prognostic effects of molecular traits of AML1-ETO. In our study, we included 254 AML1-ETO AML patients, and we found controversial results. This is probably due to the heterogeneity of the patients, difference in treatment approaches, and the effect of co-mutations.

Up to now, little information is available for contaminant mutation and predicting the prognosis of so many AML1-ETO patients in China. In this study, only c-kit mutation was able to distinguish patients with good and poor prognosis among many mutation genes concomitant with AML1-ETO. The numbers of c-kit or the presence of FLT3-ITD could not influence the OS of AML1-ETO patients. And the age, WBC count, and blast cell count are related with the OS of AML1-ETO patients. This new model may help doctors to interfere in advance to improve the prognosis and reduce the mortality of patients.

There are still some limitations in the current study. First, due to its retrospective nature, induction and consolidation regimens cannot be completely unified. Furthermore, some included patients had a high loss ratio of survival data. A larger sample size of prospective study is required to further verify the results. In this study, multi-gene sequencing and comprehensive prognostic analysis were performed for AML1-ETO-positive Chinese patients. These findings not only provided important information of the molecular structure characteristics of AML1-ETO, but they also revealed the important contaminant mutated genes with distinct clinical outcomes.

## Data Availability Statement

The sequencing data presented in our study are publicly available in the NCBI Sequence Read Archive (SRA) repository, with accession number PRJNA784000.

## Ethics Statement

The studies involving human participants were reviewed and approved by the ethics committee of the First Affiliated Hospital of Zhejiang University. The patients/participants provided their written informed consent to participate in this study.

## Author Contributions

JJ designed this study. MY, YZ, BZ, HM, WY, HT, JH and HZ collected and integrated the clinical materials. FL, SC, LL and JQ provided support for NGS. JW and CW displayed the statistical analysis of data. MY, YZ, CH, and JJ contributed to the final draft, and all authors reviewed and approved the final draft.

## Funding

This work was supported by the Zhejiang Province Natural Science Foundation of China grants (LY16H160009).

## Conflict of Interest

LL, JQ, FL, SC, and CW are employed by Acornmed Biotechnology Co., Ltd.

The remaining authors declare that the research was conducted in the absence of any commercial or financial relationships that could be construed as a potential conflict of interest.

## Publisher’s Note

All claims expressed in this article are solely those of the authors and do not necessarily represent those of their affiliated organizations, or those of the publisher, the editors and the reviewers. Any product that may be evaluated in this article, or claim that may be made by its manufacturer, is not guaranteed or endorsed by the publisher.

## Supplementary Material

Supplementary Table S1 A panel of 185 genes, which covered all the mutation hotspots of acute leukemia (AL), myelodysplastic syndrome (MDS), and myeloproliferative neoplasms (MPNs).
