# Supplementary material for: A Predictor Combining Clinical and Genetic Factors for AML1-ETO Leukemia Patients
Source: Front Oncol. 2022 Jan 14;11:783114. doi: 10.3389/fonc.2021.783114 (PMC8796117; doi:10.3389/fonc.2021.783114)
Supplement: Supplementary Table S1 — A panel of 185 genes, which covered all the mutation hotspots of acute leukemia (AL), myelodysplastic syndrome (MDS), and myeloproliferative neoplasms (MPNs). [file Table_1.docx]

| gene1 | gene2 | pValue | oddsRatio | Event |
| --- | --- | --- | --- | --- |
| CEBPA | GATA2 | 0.00000 | 32.51 | Co_Occurance |
| KRAS | NRAS | 0.0001 | 7.13 | Co_Occurance |
| WT1 | GATA2 | 0.0002 | 16.42 | Co_Occurance |
| PTEN | BCL2 | 0.0013 | 16.72 | Co_Occurance |
| GNAS | KMT2A | 0.0015 | 9.34 | Co_Occurance |
| NOTCH2 | EZH2 | 0.0026 | 12.48 | Co_Occurance |
| NOTCH2 | CUX1 | 0.0040 | 16.57 | Co_Occurance |
| CEBPA | WT1 | 0.0040 | 16.57 | Co_Occurance |
| NRAS | ASXL2 | 0.0047 | 2.80 | Co_Occurance |
| CBL | CSF3R | 0.0048 | 7.35 | Co_Occurance |
| RB1 | KMT2D | 0.0075 | 12.10 | Co_Occurance |
| EZH2 | DHX15 | 0.0086 | 4.46 | Co_Occurance |
| PAX5 | GNAS | 0.0094 | 13.29 | Co_Occurance |
| CSF3R | ASXL2 | 0.0103 | 3.28 | Co_Occurance |
| PDGFRB | GATA2 | 0.0286 | 9.68 | Co_Occurance |
| TET2 | FLT3-ITD | 0.0291 | 2.53 | Co_Occurance |
| RUNX1 | ASXL1 | 0.0325 | 4.61 | Co_Occurance |
| KDM6B | BCL2 | 0.0325 | 4.61 | Co_Occurance |
| U2AF1 | DNM2 | 0.0325 | 4.61 | Co_Occurance |
| CEBPA | U2AF1 | 0.0343 | 9.62 | Co_Occurance |
| TET2 | KIT | 0.0366 | 0.42 | Mutually_Exclusive |
| EZH2 | DNM2 | 0.0391 | 3.70 | Co_Occurance |
| CUX1 | GATA2 | 0.0456 | 8.29 | Co_Occurance |
| SETD2 | DNM2 | 0.0470 | 4.17 | Co_Occurance |
| BCL2 | NRAS | 0.0498 | 2.78 | Co_Occurance |
